# Next-generation antimicrobials: from chemical biology to first-in-class drugs

**DOI:** 10.1007/s12272-015-0645-0

**Published:** 2015-08-11

**Authors:** Michelle Lay Teng Ang, Paul Murima, Kevin Pethe

**Affiliations:** Lee Kong Chian School of Medicine and School of Biological Sciences, Nanyang Technological University, 30 Biopolis Street, #B2-15a, Singapore, 138671 Singapore; Global Health Institute, Swiss Federal Institute of Technology in Lausanne (EPFL), 1015 Lausanne, Switzerland

**Keywords:** Drug discovery, Antimicrobials, First-in-class drugs, Screening, Lead optimization, Chemical biology

## Abstract

The global emergence of multi-drug resistant bacteria invokes an urgent and imperative necessity for the identification of novel antimicrobials. The general lack of success in progressing novel chemical entities from target-based drug screens have prompted calls for radical and innovative approaches for drug discovery. Recent developments in chemical biology and target deconvolution strategies have revived interests in the utilization of whole-cell phenotypic screens and resulted in several success stories for the discovery and development novel drug candidates and target pathways. In this review, we present and discuss recent chemical biology approaches focusing on the discovery of novel targets and new lead molecules for the treatment of human bacterial and protozoan infections.

## Introduction

Since the dawn of the genomics era, the main focus of drug discovery has been on targeting cellular processes or defined enzymes that have a key role in disease pathogenesis (Hopkins and Groom 2002; Vander Heiden 2011). The target-centric approach enabled the testing of a specific biological hypothesis and the rational design of drug candidates to modulate it. Although functional genomics has transformed how antibacterial drug discovery is approached, very few genomics-driven compounds are currently in clinical development. Target-based drug discovery is overly reductionist in concept, reducing the drug–organism relationship to inordinate drug-target interplay. This dissociation from bacterial systems pharmacology is in contrast to the integrated network response to perturbation that microbes are endowed with.

Prior to the introduction of biochemical screening that emerged after sequencing of most human pathogens, drug discovery was driven empirically by a cell-based approach, abiding by the mantra of “selectively kill the bacteria first and understand why later”. These antibiotics, such as para-aminosalicylic acid (PAS) and pyrazinamide (PZA), were introduced to clinical practice with limited knowledge about the mechanism of action.

Although the field was successful in the discovery and development of the current antibacterial armamentarium (Fischbach and Walsh 2009), the limited use of empiric whole cell screening approach in recent years has contributed in part to the current dearth of new antibacterials (Sams-Dodd 2005; Fischbach and Walsh 2009; Dick and Young 2011).

A consequential impediment of empiric cell based screening is that the target protein remains unknown, making lead optimization steps more time-consuming and challenging (Plouffe et al. 2008; Swinney 2013). Nevertheless, progress in chemical biology and target deconvolution strategies has revived high-throughput whole-cell phenotypic screening (Terstappen et al. 2007), whilst exploiting cutting-edge technologies to accelerate the discovery and development of novel antimicrobials (Andries et al. 2005; Makarov et al. 2009; Rottmann et al. 2010; Pethe et al. 2013; Ling et al. 2015). This has culminated in the discovery of several first-in-class medicines with novel molecular mechanism of actions (MMOA). Automation and miniaturization of cellular screening systems enable the collation of unprecedented amounts of data for a single small molecule across a diverse collection of cellular screens, thus providing insights into a compound’s possible MMOA upon comprehensive evaluation (Plouffe et al. 2008). In fact, a recent analysis suggested phenotypic screens to be the more successful strategy for the discovery of first-in-class medicines (Swinney 2013). The rationalization for the success of phenotype-based screens was the unbiased identification of the MMOA.

Given the success of phenotypic screens in delivering new chemical entities (NCE) inhibiting virgin target space, we herein review recent chemical biology approaches focusing on the discovery of novel targets and new lead molecules for the treatment of human bacterial and protozoan infections.

## Chemical biology strategies for protozoan parasites

Protozoa are a wide group of unicellular organisms that can be found in multiple ecosystems and are often non-pathogenic for humans. However, a small subset of protozoa has evolved as lethal intracellular parasites posing serious global health challenges. Among the most deadly intracellular protozoan parasites are those of the genus *Plasmodium*, *Leishmania* and *Trypanosoma*, which are collectively responsible for millions of death every year. Due to the emergence and spread of drug resistance (Dondorp et al. 2009; Tun et al. 2015), treatment options are often limited with reduced efficacy (Wongsrichanalai and Meshnick 2008). Drug R&D have been largely inexistent for these three parasites until early 2000’ when initiatives from leading pharmaceutical industries, largely supported by philanthropic and public nonprofit organizations emerged as a new business model to develop drugs for neglected diseases. These product development partnerships (PDP) have brought promising candidates for malaria in a record time.

### Plasmodium

There are four species of Plasmodium that cause malaria in human. Collectively, they are responsible for 584,000 deaths and more than 200 million cases every year. The most common species are *Plasmodium falciparum* and *Plasmodium vivax*. The discovery and development of KAE609; an investigational drug to treat malaria is the most notable example of an antimalarial drug resulting from the effort of a PDP. The spiroindolone KAE609 was discovered in a phenotypic screen designed to identify small-molecules that rapidly clear intracellular *P. falciparum* from human red blood cells (Plouffe et al. 2008). Owing to its high potency in the cellular assay, low toxic liability and an early proof of efficacy in infected animals (Rottmann et al. 2010; Yeung et al. 2010), the spiroindolone series was optimized to improve overall properties. A reverse genetic approach was employed to identify the Na(+) efflux ATPase PfATP4 as the molecular target of the spiroindolone series (Rottmann et al. 2010; Spillman et al. 2013). Having demonstrated a remarkable efficacy in a phase 2a study at low dose, the optimized clinical candidate KAE609 has the potential to revolutionize malaria treatment (White et al. 2014; Held et al. 2015). The road is still uncertain before the introduction of KAE609 to clinical practice, but the program is a paradigm of how a PDP together with international collaborations can revolutionize the drug discovery process. Besides the discovery of KAE609, the same phenotypic screen also screen deliver KAE609, but also the novel class of imidazolopiperazines (IP) (Meister et al. 2011; Derbyshire et al. 2012), which is under clinical development (Leong et al. 2014).

A similar forward chemical genetics approach was conducted by two independent teams to identify hundreds of novel chemotypes active against drug resistant *P. falciparum* (Gamo et al. 2010; Guiguemde et al. 2010). The release of the chemical structures in the public domain is yet another example on how open innovation can accelerate drug discovery for neglected diseases. In an elegant reverse chemical genetics approach targeting 61 proteins and enzymes of *P. falciparum*, Crowther et al. identified the putative target of several promising hits using a thermal shift assay (Crowther et al. 2009). Even though validation experiments are yet to be performed to ascertain target engagement, their preliminary results set a solid foundation in spearheading translational research. Since the release of the chemical structures, several lead series have been evaluated for further evaluation (Sanz et al. 2011; Jimenez-Diaz et al. 2014; Vaidya et al. 2014). Interestingly, the promising dihydroisoquinolines compound (+)-SJ733 compound act through PfATP4 to mediate host clearance of the parasite (Jimenez-Diaz et al. 2014), which is also the target of mechanism shared with KAE609 (Spillman et al. 2013).

A most challenging subpopulation of Plasmodium to eradicate is the liver form of the disease. Plasmodium infect hepatocytes as sporozoites that first undergo a phase of maturation before emerging in the blood to cause disease manifestations. Targeting the early exoerythrocytic form of the parasite could be prophylactic since it would eradicate Plasmodium before the onset of the disease. In a technical tour de force, Meister et al. developed a high-content assay to quantify replication of *P. yeollii* inside human hepatocytes (Meister et al. 2011). An image-based approach is particularly well adapted for screening since only 1 % of the hepatocytes are infected by the parasites. By screening a collection of more than 4000 commercially available compounds that have activity against blood stage *P. falciparum*, the authors identified several chemicals that kill exoerythrocytic parasites. The most advanced drug candidate GNF179 provided protection against a challenge with *P. berghei* sporozoite, demonstrating in vivo activity against the early-liver stage of the disease. The putative target for GNF179 is pfcar1, an uncharacterized protein that is postulated to be involved in protein folding that is assumed to be essential for the biology of both the liver and blood stages of Plasmodium infection (Jonikas et al. 2009). More recently, a team at AstraZeneca utilized another high-throughput imaging assay to identify 2 more novel classes of fast-acting antiplasmodial agents; the N-aryl-2-aminobenzimidazoles, which targets the asexual blood stages of *P. falciparum* (Ramachandran et al. 2014), and the triaminopyrimidines (TAPs), which may potentially be used for single-dose treatment of malaria when used in multidrug combination therapy (Hameed et al. 2015). Exhibiting potent and wide-spectrum antimalarial activity against multiple life-cycle stages of *P. falciparum*, DDD107498 is another optimized lead derived from a 2, 6-disubstituted quinolone-4-carboxamide scaffold previously identified from a previous phenotypic screen (Baragana et al. 2015).

A key challenge in quest to eradicate malaria is the lack of potent drugs active against exoerythrocytic *Plasmodium vivax*. *P. vivax* is the most common form of malaria in South-east Asia. Although less deadly than *P. falciparum*, this species can survive in a dormant form as hypnozoites for extended period of time, leading to recurrent relapse (Wells et al. 2010). The identification of drug candidates active against hypnozoites is challenging due to the lack of predictive ex vivo models amenable to large-scale phenotypic screens. (Wells et al. 2010). Recent technical developments bring about cautious but yet resurgent optimism for the eventual discovery for an inherent cure for vivax malaria, through the complete eradication of all forms of Malaria parasites from the body. Using a low-throughput assay designed to quantify the number of hypnozoites and schizont forms of *Plasmodiumcynomolgi* (a model for *P. vivax*) inside rhesus hepatocytes, the drug candidate KAI407 was identified for activity in reducing the formation of hypnozoites (Zou et al. 2014). *In vivo* prophylactic efficacy suggests that KAI407 may indeed represent a radical cure for vivax malaria. In prospect, the recent development of transgenic fluorescent *P. cynomolgi* (Voorberg-van der Wel et al. 2013), and of platforms that recapitulate the hepatic stage of *P. vivax* (Chattopadhyay et al. 2010; March et al. 2013) will probably be instrumental in the coming year to the identification and development of drugs targeting *P. vivax* hypnozoites.

### Other kinetoplastids

Parasites of the genus Trypanosoma and Leishmania are kinetoplastid protozoan parasites that cause trypanosomiasis and leishmaniasis, respectively. These diseases, prevalent in tropical and subtropical countries, cause significant morbidity and mortality. No vaccines are available; and the current chemotherapies available for these neglected tropical diseases (NTDs) are limited with multiple shortcomings including potentially severe side effects, lengthy drug regimens and variable efficacy.

Chagas disease, caused by *Trypanosoma cruzi*, is the major cause of heart failure in Latin America. The only 2 available chemotherapies are benznidazole and nifurtimox, which have been shown to be largely ineffective and toxic, commonly causing drug resistance (Filardi and Brener 1987; Viotti et al. 2006). Drug R&D for Chagas disease is challenging due to the complex infection cycle of *T. cruzi* that can persist for many years in infected patients as trypomastigotes and amastigotes. The availability of engineered reporter gene expressing-parasites (Bettiol et al. 2009; Canavaci et al. 2010) have triggered the development of phenotypic assays suitable for HTS, as well as the establishment of new in vivo protocols (Canavaci et al. 2010; Rodriguez and Tarleton 2012) that allow faster evaluation of experimental therapeutic options.

Automated high content microscopy approaches (Engel et al. 2010; Moon et al. 2014) have been used to identify new parasitic inhibitors. To mimic the intracellular life cycle of *T. cruzi* for drug screening, Engel et al. developed and validated a flexible cell-based, high-throughput 96-well plate assay that could be used with a variety of untransfected *T. cruzi* isolates and host cells (Engel et al. 2010). This allowed the simultaneous measurement of both efficacies against the intracellular amastigote stage and host cell toxicity. Validation of the HTS assay enabled the identification of 55 hits upon screening a library of 909 bioactive compounds. Further drug testing narrowed the list down to 17 compounds that showed at least 5-fold selectivity between the inhibition of *T. cruzi* and host cell toxicity (Engel et al. 2010). Since these confirmed hits were selected from a library of clinical drugs, they could potentially be repurposed for *T. cruzi* treatment and used for mode of action and target identification studies. In a similar vein, a team from the Institut Pasteur Korea customized a high content image-based and HTS algorithm for the quantification of infection ratio and intracellular *T. cruzi* amastigote in human cell line in response to drug treatment (Moon et al. 2014). Based solely on DNA staining and single-channel images, the algorithm precisely segments and identifies the nuclei and cytoplasm of mammalian host cells as well as the intracellular parasites infecting the cells to produce various statistical parameters that can be used to assess both drug responses and compound cytotoxicity (Moon et al. 2014).

The Genomics Institute of the Novartis Foundation (GNF) has also initiated a drug discovery program for Chagas disease using phenotypic screens that measure the inhibition of proliferation of various kinetoplastid parasites to identify hits among low molecular mass compounds (Bustamante et al. 2011). To date, GNF has screened around 700,000 small molecules against the bloodstream form of *T. brucei* and the *Leishmania donovani* axenic amastigotes, which yielded around 2,000 confirmed hits in total. 44 % of these hits were also found to be active against intracellular *T. cruzi* (IC50 < 4 µM). Several of these *T. cruzi*-active compounds that possess a favorable profile have been selected for medicinal chemistry exploration with the goal of identifying lead scaffolds that could be further optimized. These early efforts have resulted in the discovery of analogs with sub-nanomolar potency against *T. cruzi* (Bustamante et al. 2011). These screens have also been recently extended to a larger 1.8 million compound library to identify *T. cruzi*-active compounds (Bustamante et al. 2011). It is expected that these screening efforts by GNF will eventually yield a single pool of anti-*T. cruzi* compounds that will be further validated and chemically optimized to yield preclinical candidates that could address this crucial gap in drug discovery for Chagas disease.

Leishmaniasis has been ranked among the most neglected of tropical diseases. As a group of diseases caused by trypanosomatids from the genus, cutaneous and mucosal leishmaniases are the predominant pathologies produced by *Leishmania* infection. Several drugs are currently available to treat cutaneous leishmaniasis, but each has limitations (Cruz et al. 2009). While target-based screening approaches for anti-leishmanial drug discovery has yielded little progress for a variety of reasons (Freitas-Junior et al. 2012), the development and implementation of HTS phenotypic assays by individual academic groups, consortia and public–private partnerships have generated several potential starting points for drug development. There is a general consensus for an urgent need for more reliable phenotypic in vitro screening that would mimic as closely as possible the definitive host environment. Therefore, genetically modified parasites expressing easily detectable reporters represent promising tools for phenotypic screening (Reguera et al. 2014).

Yet another evident advancement in this field is the development and validation of a high-content, high-throughput image-based screening assay targeting the intracellular amastigote stage of different species of *Leishmania* in infected human macrophages without the need for a reporter gene (Siqueira-Neto et al. 2012). The in vitro infection protocol was adapted to a 384-well-plate format, thus enabling acquisition of a large amount of readouts by automated confocal microscopy. This assay has enabled the screening of up to 300,000 compounds to obtain 350 hits (Siqueira-Neto et al. 2012; Reguera et al. 2014). Since the same study also established that only 4 % of the hits identified by using *Leishmania* promastigotes display efficacy against intracellular amastigote forms (Siqueira-Neto et al. 2012), intracellular amastigote-based phenotypic screening is reinforced as the most suitable approach to be developed.

An exciting approach that is midway between in vitro cell infections and experimental infections in mice consists of the use of ex vivo explants of *Leishmania*-infected organs. Target-infected organs are harvested from fluorescent or bioluminescent *Leishmania*-infected rodents for development of ex vivo explant culture. These splenic or lymph node ex vivo infected explants are advantageous over in vitro systems by including the whole cellular population involved in the host-parasite interaction: macrophages, CD3 + and CD4 + T cells, B lymphocytes and granulocytes; which could affect the therapeutic effect of the tested compound. Since a single infected spleen can yield up to four 96-well plates, the use of these ex vivo explants will also drastically reduce the number of animals used for screening, whilst still enabling medium-throughput screening capabilities. Using this approach, 4,035 compounds were screened at a single-dose concentration against luciferase-transfected *L. donovani*-infected ex vivo hamster spleens, revealing more than 200 active hits (Osorio et al. 2011). More recently, the same group has also validated a lymph node ex vivo explant model using the same bioluminescent reporter in a *L. major* strain (Peniche et al. 2014).

Furthermore, lead compounds can be scaled up to in vivo preclinical trials using rodent models of infection monitoring parasite loads by means of advanced bioimaging devices. The use of quick and reproducible fluorescent and bioluminescent readouts would greatly reduce the number of animals used for these trials and allow for an earlier stage detection of the infective process as compared with classical methods (Reguera et al. 2014). A total of near half million compounds have been screened for visceral leishmaniasis treatment through a series of cutting-edge technologies combined with optimized assays (Freitas-Junior et al. 2012). However, since more systemic approaches to develop new chemical entities for leishmaniasis have started only recently, the late discovery process is still in its infancy.

Human African trypanosomiasis (HAT), also known as sleeping sickness, is yet another example of a vector-transmitted disease caused by 2 *T. brucei* subspecies namely, *T. b. gambiense* and *T. b. rhodesiense*. Untreated HAT leads to a fatal outcome, causing significant morbidity and mortality. With no vaccine available, current chemotherapy against HAT relies on four drugs that possess several limitations and occasional severe side-effects. Whole-cell assays in HTS format for *T. brucei* are relatively new, with the development of luciferase and resazurin-based cell viability assays a 384-well format (Mackey et al. 2006; Sykes and Avery 2009a, b). HTS resazurin-based assays have been recently used to screen 87,296 compounds, resulting in 6 hits from 5 new chemical classes with activity confirmed against the causative species of HAT (Sykes et al. 2012). Being intensively used in antimalarial drug discovery as well (Smilkstein et al. 2004; Johnson et al. 2007; Izumiyama et al. 2009; Vossen et al. 2010), SYBR Green whole-cell assays, which are an indirect assessment of cell number based on quantitative detection of nuclei acids, have also been found to be applicable to *T. brucei*. To aid in drug discovery efforts for HAT, both assays were semi-automated to screen a library of 4,000 putative kinase inhibitors (Faria et al. 2015). The compounds with the most potent anti-trypanosomal activity could be grouped into 13 structural clusters. Several of the identified compounds had IC_50_ <1 μM coupled with high selectivity toward the parasite, thus providing promising starting points for lead optimization.

The limitation for the development of novel lead candidates for kinetoplastid parasites remains in target identification. Early identification of the candidate target and demonstration of target engagement would indeed accelerate drug development by using enzymatic assays or biophysical methods to rationally optimize drug candidates. While efforts can be further expanded for drug discovery for these NTDs, it is anticipated that novel drug candidates are on the horizon for the treatment of trypanosomiasis and leishmaniasis. On the model of *P. falciparum*, selection of escape mutants followed by whole-genome sequencing is probably the most straightforward approach to identify the target, or at least mechanisms of resistance.

## Chemical biology strategies for pathogenic bacteria

The emergence of multi-drug resistant (MDR) bacteria poses enormous public health issues. Despite recurrent call for actions, efforts and investments from the public and private sector do not match the threat posed by MDR germs, especially by emerging gram-negative bacteria, often referred to as superbugs. Among the most deadly bacteria are *Mycobacterium tuberculosis*, the etiological agent of human tuberculosis, and those known as the “ESKAPE” pathogens *Enterococcus faecium*, *Staphylococcus aureus*, *Klebsiella pneumoniae*, *Acinetobacter baumanii*, *Pseudomonas aeruginosa*, and *Enterobacter species* that cause the lion’s share of hospital-acquired infections (Rice 2008; Boucher et al. 2009; Rice 2010). The global R&D pipeline remains extremely thin for broad-spectrum antibiotics, especially for the gram-negative ESKAPE bugs (Boucher et al. 2013). Innovation in chemical biology and target deconvolution strategies represents the most promising approach to discover novel antimicrobials, as exemplified by recent success stories for tuberculosis.

### Mycobacterium tuberculosis

Tuberculosis (TB) remains a major global health challenge, with nearly 1.5 million TB-related deaths in 2013 alone (WHO 2013). Underlying the endemic is the emerging epidemic of multi-drug resistant (MDR-TB) and extensively-drug resistant (XDR-TB) TB strains. With dwindling treatment options for MDR and XDR-TB, one of the pertinent key issues faced by the TB research community is the daunting challenge of discovering and developing new anti-TB drugs. Phenotypic screens have been used extensively in the last decade to discover innovative drug candidates, contributing to replenish the drug pipeline.

Historically, most anti-tuberculosis drugs were identified by screening candidate drugs against *Mycobacterium tuberculosis* (Mtb) replicating in culture broth medium. The exception is pyrazinamide, a sterilizing first-line anti-TB drug that was discovered by a bold phenotypic screen in infected animals (Malone et al. 1952). Mode of action was usually not understood at the time of introduction to clinical practice. The sequencing of the Mtb genome in 1998 (Cole et al. 1998) led to the emergence of target based drug discovery and prompted the field to reconsider the utility of phenotypic screens. The difficulty in finding targets inhibited by the compounds with whole cell activity was central to the departure from the phenotypic screening paradigm. Nevertheless, the landscape changed rapidly. Improved technologies for de-orphaning phenotypic screening hits has prompted renewed interest in pursuing whole cell phenotypic screens for novel, constructive sources of lead molecules/series.

The success of chemical biology for TB drug discovery is best epitomized by the discovery of the diarylquinoline bedaquiline (Sirturo^®^) (Andries et al. 2005). Bedaquiline is the first drug approved for the treatment of tuberculosis in the last 40 years. The drug was identified from a corporate collection screening campaign against *Mycobacterium smegmatis*, a surrogate environmental, non-pathogenic mycobacteria (Andries et al. 2005). Limited chemical optimization of a diarylquinolone series led to bedaquiline, a compound with exceptional potency against Mtb in vitro and in animal models (Andries et al. 2005). Whole-genome sequencing of spontaneous mutants resistant to bedaquiline revealed the F_0_F_1_ ATP synthetase as the drug target. Despite its evolutionary conservation, bedaquiline is highly specific for the mycobacterial complex. The inhibition of ATP synthase leads to ATP depletion and pH homeostasis imbalance, triggering bacterial death of both replicating and non-replicating mycobacteria (Deckers-Hebestreit and Altendorf 1996; Rao et al. 2001). Bedaquiline recently underwent accelerated approval by the Food and Drug Administration (FDA) for the treatment of MDR and XDR TB (Cohen 2013).

Delamanid is another drug available for the treatment MDR-TB that was granted conditional approval by the European Medicine Agency late in 2013 (Barry 2015). Full safety and efficacy is currently being evaluated in Phase III clinical trials. Delamanid was optimized from a series of highly potent nitro-imidazole (Stover et al. 2000) that inhibits mycobacterial growth by targeting multiple essential processes, including mycolic acid synthesis and respiration (Stover et al. 2000; Matsumoto et al. 2006; Singh et al. 2008). PA-824, another clinical phase nitro-imidazole drug candidate discovered by whole-cell screening, showed the promise to shorten the time of MDR-TB therapy when given in combination with moxifloxacin, pyrazinamide and/or bedaquiline (Diacon et al. 2012; Dawson et al. 2015; Tasneen et al. 2015).

Using a similar approach, ppromising drug candidates with a novel mode of action were recently reported. Of particular interest is a series of indolcarboxamine that inhibits mycobacteria growth by interfering with the function of MmpL3, a transporter of trehalose monomycolate that is essential for mycobacterial cell wall biosynthesis (Rao et al. 2013). The optimized indolcarboxamine drug candidate NITD-304 and NITD-349 have excellent in vivo efficacy, low toxicity and favorable pharmacokinetic properties in multiple species, which are properties that support clinical development of the series for the treatment of MDR-TB. The same team recently reported on the discovery of yet another promising class of mycolic acid inhibitors, the 4-hydroxy-2-pyridones (Manjunatha et al. 2015). This novel chemical series inhibits InhA, an essential enzyme involved in mycolic acid synthesis (Takayama et al. 1975), which was also recently established as the target of the natural compound pyridomycin (Hartkoorn et al. 2012, 2014). GSK has also released a set of 177 potent non-cytotoxic hits active against Mtb using comparable screening methodologies with the aim of fuelling open-source early-stage drug discovery activities (Ballell et al. 2013). The set of compounds represent a valuable resource for the TB community to initiate lead optimization and/or target identification programmes.

By virtue of being a facultative intracellular bacterium that becomes partially phenotypically drug resistant to multiple antibacterial when hiding inside macrophages, an attractive approach to circumvent the limitations of in vitro culture broth media is to screen for compounds that kill Mtb replicating inside macrophages (Kumar et al. 2010). Indeed, the nutrients and metabolites that Mtb use to replicate and survive in eukaryotic cells is largely predictive of the in vivo situation, making it an attractive platform to discover novel classes of anti-TB drugs. Several teams have reported on the development of systems to screen for drugs or siRNA that interfere with the survival of Mtb inside macrophages (Christophe et al. 2010; Sundaramurthy et al. 2013).

Using automated confocal fluorescent microscopy to monitor the intracellular growth of GFP-expressing Mtb H37Rv, a team from the Institut Pasteur Korea developed a rapid phenotypic assay to screen large chemical libraries in 384-well format (Christophe et al. 2009). This phenotypic HTS assay was the first successful demonstration for the feasibility of large scale screens against intracellular mycobacteria. A series of dinitrobenzamide derivatives (DNB) targeting the decaprenyl-phosphoribose 2′ epimerase DprE1/DprE2, thus blocking arabinogalactan synthesis, was discovered on this platform (Christophe et al. 2009). Interestingly, an independent study validated further the decaprenyl-phosphoribose 2′ epimerase as an attractive drug target (Makarov et al. 2009).

The benefit of the macrophage platform was further ratified with the discovery of the optimized imidazopyridine amide (IPA) drug candidate Q203 (Pethe et al. 2013). Q203 is the lead drug candidate that was selected after an evaluation of 477 derivatives (Kang et al. 2014). The initial discovery of the anti-TB activity of the IPA series was reported in 2011 (Moraski et al. 2011). Q203 displayed potent inhibitory growth against MDR and XDR Mtb clinical isolates in vitro by inhibiting the function of the respiratory chain, a mechanism shared with the drug bedaquiline. Genetic and biochemical evidences pointed to the mycobacterial cytochrome bc-1 as the molecular target of the IPA series (Abrahams et al. 2012; Pethe et al. 2013). Q203 combines favorable properties including bactericidal activity in the mouse model of tuberculosis at low dose and favorable pharmacokinetic with safety profiles, making this compound a promising drug candidate for tuberculosis. Intriguingly, scientists at EPFL have identified the existing drug lansoprazole as an antituberculous prodrug that also targets cytochrome bc-1 in a host cell-based high throughput screen as well, albeit using lung fibroblasts (Rybniker et al. 2015).

Recently, another large-scale compound screen in infected macrophages revealed several interesting compound series that represses the intracellular Mtb growth by inhibiting mycobacterial cholesterol metabolism (VanderVen et al. 2015). Since cholesterol is a carbon source used by Mtb in macrophages and in vivo (Rohde et al. 2012) but not in vitro, these interesting compounds series could not have been identified by screening in classical culture broth media, illustrating the benefit of using relevant models for bacterial phenotypic screen.

The added benefit of screening for anti-TB drugs in eukaryotic cells opens the possibility of identifying anti-virulence drugs (Rybniker et al. 2014) or host-targeted drugs that stimulate cellular pathway critical for the maintenance of the infection (Sundaramurthy et al. 2013; Stanley et al. 2014).

Finally, the development of a rapid screening platform for anti-TB agents in whole animals represents a very attractive approach. Two systems were recently developed using *Mycobacterium marinum* as a surrogate for Mtb. Being a natural fish pathogen, the infection process in zebrafish larvae recapitulates many aspects of tuberculosis pathogenesis in humans (Swaim et al. 2006). The zebrafish larvae platform was developed in 96 well plates and relies on automated fluorometric in situ measurement of drug efficacy and toxicity. Since the entire procedure is performed in 96 well-plates, numerous drugs can be evaluated in parallel. The assay was validated using known direct- and host-acting drugs (Takaki et al. 2012, 2013). More recently a comparable screening approach was optimized in amoeba (Kicka et al. 2014). The assay was developed in the amoeba *Acanthamoeba castellanii* infected with *M. marinum* and validated with several known anti-TB drugs. In principle, the system is suitable to identify host-targeted drug candidate and compounds interfering with virulence, which represent an advantage compared to classical screening approaches. The performance and benefits of such platforms remain to be determined in a large-scale compound screening campaign.

### Broad spectrum antibacterials

#### Gram-positive bacteria

Antibiotic resistance has become a major problem in the treatment of Gram-positive bacterial infections, with some of the most important gram-positive resistant organisms including penicillin-resistant *Streptococcus pneumonia* and methicillin-resistant *Staphylococcus aureus* (MRSA) (Klevens et al. 2007; Arias and Murray 2009). Lately, a number of contemporary and prominent approaches have produced promising data in the quest for broad-spectrum bactericidal antibiotics that could improve the clinical outcomes for the treatment of Gram-positive bacterial infections.

The first notable study involved a cell-based screen of 1280 bioactive compounds from the Library of Pharmacologically Active Compounds (LOPAC) library against a constitutively-expressing luciferase Mtb strain under nutrient-deprivation conditions in order to seek inhibitors with activity against replicating and nonreplicating Mtb (Harbut et al. 2015). This screen culminated in the unexpected identification of auranofin, an orally bioavailable FDA-approved anti-rheumatic drug, as possessing potent bactericidal activities against a number of other clinically important Gram-positive bacterial species. Auranofin exerts its effects through a unique mechanism involving the inhibition of the bacterial thioredoxin reductase, a protein essential in many Gram-positive bacteria for maintenance of the thiol-redox balance and protection against reactive oxygen species (Scharf et al. 1998; Uziel et al. 2004). These findings not only suggest auranofin as a viable candidate worth repurposing for antibacterial therapy; but its associated universal mode of action also highlight the prospects of targeting the thioredoxin-mediated redox cascade of Gram-positive pathogens for the development of novel broad-spectrum antibacterials.

Another significant breakthrough is the discovery of novel antibiotics identified from soil bacteria recalcitrant to grow under laboratory conditions (Ling et al. 2015). The development of several meticulous methods to grow uncultured organisms via in situ cultivation (Kaeberlein et al. 2002; Nichols et al. 2010) or by using specific growth factors (D’Onofrio et al. 2010) hold great promise to identify novel antibiotics from natural sources. The multichannel iChip (Nichols et al. 2010) device was designed to isolate and grow uncultured bacteria in diffusion chambers in situ, enabling the identification of numerous novel soil bacteria. A large scale screen of secreted secondary metabolites from 10,000 bacterial isolates against *S. aureus* led to the discovery of the novel antibiotic teixobactin. Teixobactin is an unusual depsipeptide that displays astounding activity against Gram-positive pathogens, including experimentally proven in vivo efficacies against drug-resistant pathogens in a number of animal models of infection (Ling et al. 2015). Due to its unusual mode of action involving multiple targets and binding to a highly conserved motif of lipid II and lipid III to inhibit peptidoglycan synthesis, teixobactin has been construed as a promising therapeutic candidate with favorable pharmacokinetic parameters (Ling et al. 2015). Primarily, the results of this pioneer study suggest that additional natural compounds with similarly low susceptibility to resistance are present in nature and may potentially be discovered following a similar approach.

#### Gram-negative bacteria

Gram-negative bacteria are best defined by their ability to acquire and transfer drug resistance genes to other bacteria (Hall and Collis 1998); along with their characteristic cell envelope which comprise of a unique outer membrane of lipoproteins, β-barrel proteins, lipopolysaccharides and phospholipids, and an inner membrane composed of a phospholipids bilayer. Compounding the problem of antimicrobial-drug resistance is the immediate threat of a reduction in the discovery and development of new antibiotics for the treatment of Gram-negative bacteria (Boucher et al. 2009). Accordingly, there is an urgent need for atypical, originative and alternative modes of drug discovery veering away from the conventional target-based approach for drug screening.

One such notable approach was the utilization of the Pfizer compound library consisting of ~1.6 million compounds in an unbiased whole-bacterial cell screening for growth inhibition of a membrane-compromised, efflux pump-deficient strain of *E. coli* (*tol C, imp*) (Miller et al. 2009). This radical methodology focused on targeting an antibacterial space resembling those of eukaryotic targets, thus facilitating the identification of a series of antibacterial pyridopyrimidines derived from a protein kinase inhibitor pharmacophore. These compounds were later found to possess a previously undescribed MMOA for antibacterial activity by targeting the ATP-binding site of biotin carboxylase (BC), which catalyzes the first enzymatic step of fatty acid biosynthesis (Cronan and Waldrop 2002). Remarkably, these BC inhibitors also exhibited outstanding potency against clinical isolates of fastidious Gram-negative pathogens including *H. influenza* and *M. catarrhalis*; causative agent of many respiratory tract infections. Despite the structural similarities of the BC active site to those of eukaryotic protein kinases, inhibitor binding to the BC ATP-binding site was found to be disparate from the protein kinase-binding mode with the inhibitors, displaying selectivity for bacterial BC. The implications behind the identification of antibacterials derived from a protein kinase inhibitor pharmacophore suggest that the huge array of eukaryotic inhibitors present in these pharmaceutical libraries could be mined for their activity against structurally related bacterial targets such as protein kinases involved in cell–cell signaling lipopolysaccharide sugar kinases involved in Gram-negative cell wall formation (Miller et al. 2009).

In a similar manner, the AstraZeneca compound collection (~1.2 million compounds) was screened in 384-well plate format against a permeabilized *E. coli* strain (W3110 ∆*waaP*) (McLeod et al. 2015). The shortened lipopolysaccharide chain in this modified strain increased membrane permeability to small molecules and warranted the discovery of novel pyridinemidazole compounds that inhibited lipoprotein trafficking from the inner to the outer membrane. Subsequent studies via resistance mutation mapping and biochemical transport assays further demonstrated the inhibition of function of the lipoprotein-releasing system transmembrane proteins (LolCDE complex) via these compounds. Being the first reported inhibitors of the LolCDE complex, this novel compound class not only displayed a unique and specific mechanism of inhibition for Gram-negative bacteria, but the identification of their associated novel drug target suggest further exploitation of the outer membrane lipoprotein transport pathway as a target for antimicrobial therapy.

Although phenotype-based screens have expanded the repertoire of new potential drug candidates, one of the biggest impediments is unraveling the relationships between the phenotype(s) caused by biologically active small molecules and their respective mechanisms (Burdine and Kodadek 2004). Moreover, due to the poor predictive value of conventional in vitro culture conditions used to analyze drug candidates (Garber 1960; Brown et al. 2008; Brinster et al. 2009; Pethe et al. 2010), it is necessary to innovate alternative means to characterize the MMOA of biologically active molecules. A more unconventional approach has been established to identify inhibitors of *E. coli* grown under nutrient limitation. Employing metabolomics, the authors demonstrated the promise of metabolite suppression profiling as a novel method for assigning a possible mode of action for metabolic inhibitors that are frequently identified in phenotypic-based screens (Zlitni et al. 2013). This inventive strategy led to the discovery of novel inhibitors of glycine and biotin biosynthetic pathways in *E. coli* and outlined a novel platform for the identification of small-molecule inhibitors of essential metabolic pathways that would be potentially applicable and easily adapted for most bacteria.

Nonreplicating and metabolically quiescent bacteria are causal for latent infections and relapses following ‘‘sterilizing’’ chemotherapy (Manina et al. 2015). Actively shifting the characteristic metabolic flux of drug-tolerant non-growing but metabolically active (NGMA) microbes toward an actively growing state offers a fresh prospect to control such populations. The notion of metabolic modulation to enhance the efficacy of current antibiotics provides key insights into the Achilles’ heel of persistent cells (Murima et al. 2014). This was recently demonstrated by Kim et al. with the unprecedented approach of screening a compound library against persistent *E. coli* in combination with ampicillin in a bid to identify specific killers of bacterial persisters (Kim et al. 2011). A small polycyclic molecule C10 was shown to sensitize bacterial persisters to ampicillin killing. Although the detailed mode of action for C10 remains undeciphered, such rudimentary yet elucidative studies capitalizing on the use of single-chemical supplementation could be applied on a universal basis to other bacterial systems to discover inhibitors targeting NGMAs (Wakamoto et al. 2013). Other potential metabolic strategies for eliminating persister cells include collapsing the proton motive force (Rao et al. 2008; Allison et al. 2011; Farha et al. 2013; Peng et al. 2015), dissipating intracellular nutrient storage in mycobacteria (Baek et al. 2011), or inducing reactive oxygen species potency of bactericidal antibiotics (Kohanski et al. 2007).

## Future prospects for chemical biology screens

To support the discovery of novel antimicrobials, it is heartening to observe how public and private initiatives have collaborated, thereupon allowing researchers to gain access to vast collections of bioactive compounds for drug screens. This avenue has provided further motivation for more researchers to concert towards the identification of new leads, expanding efforts in the drug discovery sector. Several of the chemical biology approaches discussed in this review have proven to be quintessential for detecting small-molecules with optimal selective indexes for specified pathogens that usually warrant favorable results in a preclinical model of the disease; avoiding the nuances encountered in target-based screenings associated with poor permeability or degradation into inactive metabolites (Reguera et al. 2014).

Indeed, chemical biology screens have proven to be effective for the discovery of antimicrobials, as evidenced by the discovery of a number of potential and validated first-in-class drugs associated with novel drug targets/pathways emerging from these screens. In terms of drug discovery for infectious diseases, chemical biology screens appear to have gained the most traction and advancements in the fields of malaria and Mtb likely due to the motivation behind relevant interest groups, but have also proven to be applicable to other human pathogens as well (Table 1). Furthermore, many of the concepts behind chemical biology approaches discussed in this review have the potential to be adapted to other human pathogens with some prudent remodeling. Due to the limited chemical diversity in screening libraries (Payne et al. 2007), it is also plausible to widen the scope of these screens to include metabolism curated libraries and natural products, which have shown potential in pilot studies (Miller et al. 2009; Ling et al. 2015). Additionally, chemical biology screens have advanced by quantum leaps in terms of technology, having progressed from simplistic in vitro whole cell screens to the incorporation of reporter genes for bioluminescent, fluorescent or enzymatic measurements via HTS platforms, and more recently, to bioimaging readouts in ex vivo explants. Ultimately, the development of large-scale in vivo chemical biology screening platform, with cutting edge imaging and target deconvolution strategies, certainly has the potential to accelerate even further antimicrobial drug discovery.

**Table 1:** Novel chemical classes identified through chemical biology screens

Disease	Chemical class	Lead compound(s)	Chemical structure	Identified target	MMOA	Key reference
Malaria	Spiroindolones	KAE609		PfATP4	Disrupts intracellular sodium homeostasis	Rottmann et al. (2010)
	Dihydroisoquinolines	SJ733		PfATP4	Disrupts intracellular sodium homeostasis	Jimenez-Diaz et al. (2014)
	Imidazolopiperazines	GNF179		Pfcar1	Unclear	Meister et al. (2011)
	Imidazopyrazines	KAI407		PI(4)K	Alters the intracellular distribution of phosphatidylinositol-4-phosphate	Zou et al. (2014)
	N-aryl-2-aminobenzimidazoles	Compound 12 in Ramachandran et al. (2014)		NA	Unclear	Ramachandran et al. (2014)
	Triaminopyrimidines	Compound 12 in Hameed et al. (2015)		Unclear	Unclear	Hameed et al. (2015)
Tuberculosis	Diarylquinolones	Bedaquiline		F_0_F_1_ ATP synthetase	ATP depletion and pH homeostasis imbalance	Andries et al. (2005)
	Nitro-imidazoles	Delamanid PA-824		Unclear	Inhibits mycobacterial growth by targeting multiple essential cellular processes, including protein, mycolic acid synthesis and respiration	Stover et al. (2000)
	Indolcarboxamines	NITD-304		MmpL3	Inhibits mycobacterial cell wall synthesis	Rao et al. (2013)
		NITD-349				
	4-hydroxy-2-pyridones	NITD-916		InhA	Inhibits mycolic acid synthesis	Manjunatha et al. (2015)
	Dinitrobenzamides	NA	NA	DprE1/DprE2	Blocks arabinogalactan synthesis	Christophe et al. (2009)
	Imidazopyridine amides	Q203		bc-1 complex	Inhibits ATP synthesis	Pethe et al. (2013)
	Pyridomycins	Pyridomycin		InhA	Blocks both the NADH cofactor– and lipid substrate–binding pockets of InhA	Hartkoorn et al. (2012)
Others	Depsipeptide	Teixobactin		Multiple targets	Inhibits peptidoglycan synthesis	Ling et al. (2015)
	Pyridopyrimidines	NA	NA	biotin carboxylase (BC) complex	Inhibits fatty acid synthesis	Miller et al. 2009
	Pyridinemidazoles	NA	NA	LolCDE complex	Inhibits lipoprotein trafficking from the inner to the outer membrane	McLeod et al. (2015)

*NA* Not available
